# A brief universal parenting program for recently settled immigrants in Sweden: a feasibility study

**DOI:** 10.1186/s40359-026-04026-2

**Published:** 2026-01-26

**Authors:** Maja Västhagen, Metin Özdemir, Birgitta Kimber, Ata Ghaderi, Vanessa Place, Pia Enebrink

**Affiliations:** 1 Department of Clinical Neuroscience, Karolinska Institutet, Nobels väg 9, Stockholm, 171 77 Solna Sweden; 2 School of Behavioural, Social and Legal Sciences, Örebro University, Örebro, Sweden; 3 Department of Clinical Science, Umeå University, Umeå, Sweden

**Keywords:** Parenting program, Forced migration, Resilience, Promotion

## Abstract

**Abstract:**

The accumulative migration stressors and challenges of parenting adolescent children in a new cultural context indicate a need for culturally sensitive parenting programs to support parents with immigrant backgrounds.

**Objective:**

This study evaluated preliminary outcomes, acceptability, and feasibility of a new brief universal 4-session universal parenting program delivered in mother tongue. The aim is to strengthening parenting skills and ultimately resilience among recently settled immigrant families.

**Methods:**

A convergent mixed methods pre-, post-test study design was applied. Paired *t*-tests were used to evaluate the preliminary effectiveness based on parental self-report on various aspects of the parent-child relationship, parental strategies, efficacy to support the child’s schooling, sense of belonging to the larger society, as well as hopefulness. Further, session reports from parents and session-reports and semi-structured interviews with group leaders were used to explore the feasibility and acceptability of the intervention. The interviews were analysed using content analysis.

**Results:**

A total of 53 recently settled parents (7 groups) in Sweden speaking Arabic, Dari, or Somali with at least one teenager aged 12–16 years took part in the program. Parental perceived ability to constitute a safe haven in their relationship to their child (*t*(282) = 2.0, *p* = .047) and their perceived societal belonging (*t*(285) = 4.60, *p* = < 0.001) increased significantly over time. Most other changes were in expected direction, but did not reach level of significance. Parents found the program being meaningful and were willing to recommend it to other parents. The qualitative results based on interviews with group leaders resulted in one overall theme, *Meeting parents where they’re at and building trust*, with three subthemes: *Components of success; Learning together*, and *Access.* The integration of the quantitative and qualitative results suggested importance of creating circumstances that lead to a ‘brave space’, i.e., where participants can express themselves and learn from each other.

**Conclusion:**

The program seems acceptable and feasible for parents and group leaders. Cultural bridges for participation and recruitment, as well as ample time for relation-building seems crucial. These results lend support to conducting a randomized controlled trial to investigate the effectiveness of the intervention.

**Trial registration:**

Retrospectively registered at ISRCTN (10645626) on 17th February 2025.

**Supplementary Information:**

The online version contains supplementary material available at 10.1186/s40359-026-04026-2.

The number of migrants continues to increase worldwide. In 2020 the number of international migrants was 281 million [1] and in 2024 the number of asylum-seekers and refugees was almost 50 million [2]. In relation to previous migratory experience, the post-migration context can be an equally powerful determinant of mental health [3], where different daily stressors have strong associations with poor mental health [4].

One stressor may be navigating parenthood in a new cultural context. Families from an immigrant background have the pressure of establishing themselves in a new society and cultural context [5]. Refugee parents in Sweden describe a feeling of disempowerment in their identity as a parent, due to challenges related to unemployment, financial constraints, language barriers and unfamiliarity with the society at large [6, 7]. Further, many immigrant parents describe more authoritarian or hierarchical parenting strategies in their home country whereas egalitarian parenting is emphasized in Sweden [8]. They also describe how unfamiliarity with the local parenting norms and legislation leads to fear and a passive parenting style [6]. Youth tend to adapt relatively quickly to the new culture, whereas it often takes more time for parents who immigrated as adults [9]. This discrepancy might create an acculturation gap [6, 8, 10]. Although parents express both gratitude for being able to come to Sweden, they also acknowledge a need for more support in the form of support programs or advice for handling the acculturation gap and to navigate family relations in the new cultural context [8, 10–12].

Despite the challenges and stressors that immigrant parents encounter in the receiving context, they can be exceptionally resilient. There is no agreed definition of psychological resilience, and the concept has evolved through time [13]. A system-orientated definition suggests: “the capacity of a system to adapt successfully through multisystem processes to challenges that threaten the function, survival, or development of the system” [14, p. 524]. Factors of resilience within a family can include sensitive caregiving, close relationships and social support, sense of belonging, hope, positive views of self, family or group, and motivation to adapt (for a full list, see 14). These factors are suggested to operate across different systems, such as the individual, family, school, community or cultural group [14]. Further, culture permeates every aspect of resilience, not at least parenting practices [14].

One way to support and strengthen immigrant parents could be to offer parenting programs. These programs aim to empower parents through bolstering desirable parenting behaviours and the relationship between parent and youth [15, 16]. However, few parenting programs are available for forced migrant parents [17] and immigrant parents [18] despite promising effects in promoting positive parenting practices and psychological health among forced migrant families [17]. It has been suggested that parenting programs could have a potential to mitigate familial displacement [19]. Parenting programs seem to be an underutilized resource to promote positive development for families in precarious situations, such as forced migration [17]. Such interventions should in theory be both acceptable and feasible, in that people receiving or delivering the intervention consider and experience it as appropriate (compare[20]) and possible to conduct [21].

A new brief universal parenting program was therefore developed. We targeted parents of adolescents, since adolescence can be a particularly challenging period for a family in general [22], and for immigrant families in particular [14]. The program was based on previous research and qualitative data from interviews in mother tongue with newly arrived parents of adolescents [10]. The findings from this qualitative study suggested that the recently arrived parents of adolescents displayed high motivation and hope to become part of the new society, learn the new language, support their youth, and find ways to navigate and adjust parenthood in the new cultural context. Parents emphasized the importance of both keeping heritage culture *and* engaging in the new culture, as well as a need for new ways of being a parent, and for bridging over the acculturation gap between the parent and youth.

The overarching aim of the new program is to strengthen and promote resilience in a family through a healthy and strong parent-child relationship. The adolescence period is suggested as a period for consolidating the parent-child relationship and attachment [23] hence why parental communication strategies and skills might need to be strengthened to maintain positive relationships with the adolescent child. Building on knowledge from earlier studies, we wanted the program to encourage parents to navigate parenthood in relation to the Swedish society (compare 8, 10) and to function as a social platform (compare 18). Earlier studies have also suggested that parents request culturally informed interventions tailored to their specific needs [7], which may improve engagement and acceptance [18]. Therefore, we strived for a culturally sensitive intervention through running the program in mother tongue with culturally competent group-leaders.

## Objective and research questions

This study addressed the preliminary outcomes, acceptability and feasibility of a new parenting program, focusing on strengthening resilience among recently settled immigrant parents. A convergent mixed methods pre-post design was used, a type of design in which qualitative and quantitative data are collected in parallel, analyzed separately, and then merged. The emphasis was put on the quantitative parts (QUANT + Qual). The research questions were:

- What are the preliminary effects of the program on parent-child relationship, parenting strategies, societal belonging, efficacy to support the child’s schooling and hopefulness?
- How do recently settled immigrant parents perceive participating in the program?
- How do the group-leaders experience the program?

## Method

### Design

The non-controlled pilot feasibility study used a convergent mixed method, pre- post-test study design, and was conducted during 2022–2023. The preliminary evaluation of outcomes was based on parental self-report. Session reports from parents and group-leaders were used to explore the feasibility and acceptability of the intervention. Semi-structured interviews with the group-leaders, together with short diaries after each session, deepened the understanding by exploring experiences of conducting the program. The trial was registered at ISRCTN (date 17/02/2025, number 10645626).

### Participants and setting

#### Parents

Somali, Kurdish, and Arabic speaking populations comprised the largest recently settled immigrant and refugee groups in Sweden [24] during the planning stage of the project. An update before the actual start of the project showed that Dari and Tigrinya should also be added as language groups to the project. Immigrants in these language groups in Sweden are mainly forced migrants, i.e. asylum-seekers, refugees but also people migrating for family reunification [25]. The study was conducted in the Stockholm region. Inclusion criteria were being parent with at least one teenager (12–16 years old), speaking one of the target languages and having arrived in Sweden within the past 6 years, as acculturative changes tend to occur during the first 6–7 years in a new cultural context [26]. The aim was to reach a sample size between 70 and 120 to reliably detect pre- to post-test differences for effect sizes in the range of *d* = 0.25 and 0.30 with a power of 0.80 and *p* <.05 (one-tailed *t*-test [27]. Nevertheless, the research team managed to include 53 participants in the pilot study.

#### Group-leaders

Leaders who were native speakers of one of the target languages were recruited via service centres for immigrants, family centres, a cultural doula organization, among interpreters, and language schools for immigrants. All group-leaders had experience of working with the target population and in a group setting. Most of the leaders had themselves arrived in Sweden as refugees. The leaders received a three-day training which included information on the research project, the theoretical underpinnings of the parent training program, how to lead each session, conduct roleplays, lead discussions, and training in how to use communication skills as a group-leader. In total, 11 group-leaders ran the parent training groups, 8 women and 3 men. The leaders had various occupations including teachers, interpreters, coaches, cultural doulas, integration pedagogue, assistant nurse and vocational teacher. Their mean age was 49 years. Eight of them had led parent training groups before.

### Procedure

Several recruitment strategies were applied. Information about the study was spread through municipality centres, language cafés, cultural associations, Swedish for immigrant classes, internet platforms and letters to all recently settled families in a certain area of Stockholm. We used translated flyers and had interpreters when informing parents about the study. Often the group leaders took part in the recruitment meetings as interpreters and cultural brokers. Different locations were used for running the parenting groups, including schools and community centres. Sessions were scheduled flexibly, mornings, evenings, weekends, or school hours, depending on site needs and family schedules. They were held in local venues close to participants’ homes to reduce barriers and encourage engagement.

We aimed for 8–10 parents in each group, and to have at least three parenting groups in each language. When parents were recruited, an information meeting was arranged where the parents filled in informed consent forms and responded to the pre-test measures before the intervention. Parents who could not read or write received help in reading the items and a whiteboard was used to illustrate the response options when needed. Parents completed the post-test assessment directly after the last session. Session reports were answered at the end of each session by both leaders and parents. Interviews were conducted with all group-leaders (*n* = 11) in Swedish through telephone.

### Measures

Quantitative data was collected through self-report measures before and after the intervention.

*Parent Network of Relationship Inventory Behavioral Systems Version (NRI-BSV;* [28]. The measure assessed parent-child relationship quality using a 5-point Likert scale, (1 = never, 5 = very often), with a higher score indicating a stronger bond. The NRI-BSV has shown good psychometric properties [28]. Three subscales were used in this study (each consisting of 3 items): Companionship, Safe haven, and Secure base. The Companionship subscale measures behaviours that indicate a connection between the parent and child. An example question is “How often do you and your child have fun together? In the present study the Cronbach’s alpha for internal consistency of the companionship subscale was α =.62 at pre-test and α =.82 at post-test. The Safe haven subscale refers to the extent to which the child seeks security through the parent when distressed. An example question is ”How much does your youth turn to you for comfort and support when he/she is troubled about something?”. The Cronbach’s alpha was α = 0.74 at pre-test and α = 0.89 at post-test. The Secure base subscale measures the extent to which the parent offers a secure base from which the child can explore behaviours outside of the parent-child relationship. An example question is “How often do you encourage your child to try something new that they want to do but feel nervous about?”. Cronbach’s alpha was α = 0.66 at pre-test and α = 0.91 at post-test.

*Warmth and conflict.* Measures warmth and conflict in the family based on 9 items earlier used to evaluate Family Check-up (FCU) (e.g [29]). The *Warmth* subscale consists of five items from the Adult-Child Relationship Scale (ACRS), which is a modified version of the school-based Student-Teacher Relationship Scale (STRS; [30]). The STRS has demonstrated good reliability and validity across multiple studies [30–32]. Parents answered questions such as: “My child openly tells me about their feelings about different things” on a 5-point Likert scale (1 = definitely not, 5 = definitely), with higher scores corresponding to greater warmth. The Cronbach’s alpha for the *Warmth s*ubscale was α = 0.90 at pre-test and α = 0.94 at post-test. The *Conflict* subscale consists of 4 items adapted from the PAL2 project at the University of Oregon Child and Family Center. The subscale included statements such as “My child got what he or she wanted by getting angry”. The items were rated on a 7-point Likert scale (1 = never, 7 = more than 7 times), with higher scores corresponding to higher levels of conflicts during the past month. The Cronbach’s alpha for the *Conflict* subscale was α = 0.70 at pre-test and α = 0.77 at post-test.

*Validation.* This measure is a five-item scale capturing validation in a parent-teenager dyad. The parent answers questions such as “My teenager can express vulnerability and at the same time receive support”, rated on a 3-point Likert scale (1 = completely correct, 3 = completely incorrect). This measure was developed for use in another study with families with teenagers [33]. The internal consistency for the *Validation* scale in this study was α = 0.77 at pre-test and α = 0.94 at post-test.

*The Societal Belongingness Scale.* This scale was developed to measure the feelings of being a connected, affiliated, and a respected member of the larger society [34]. Parents answers 5 questions, such as “I feel that I am a part of the Swedish society” on a 5-point Likert scale (1 = do not agree at all, 5 = totally agree), with higher scores indicating a stronger sense of belonging. An initial evaluation of the measurement in an immigrant youth population in Sweden [34] demonstrated evidence of measurement invariance and concurrent validity. An exploratory factor analysis of the measure with Promax rotation suggested that the negatively worded item (i.e., I feel like I am an outsider here in Sweden) loaded on a separate factor itself with low cross-loading on the factor where all other items loaded on. Thus, this item was not included in creating the scale score. The internal consistency of the 4-item Societal Belongingness Scale was α = 0.72 at pre-test and α = 0.87 at post-test.

*The parental Sense of Efficacy for Helping the Child Succeed in School.* The 7-item version of the Parental Sense of Efficacy for Helping the Child Succeed in School [33] was used to assess parents’ beliefs of their ability to be involved in children’s schooling. The measure has demonstrated predictive validity in that parents’ efficacy beliefs predicted their involvement in school- and home-based activities related to their children’s education [35]. The items (e.g., I feel successful about my efforts to help my child learn) were rated on a 4-point Likert scale (1 = strongly disagree, 4 = strongly agree). The negatively worded items were reversely coded, and the scores were averaged to create a scale score. The internal consistency was α = 0.70 at pre-test and α = 0.79 at post-test.

*Hopefulness.* This scale was developed for the Youth and Diversity Project [36] and consists of five items targeting parents’ hopefulness for the future. A factor analysis indicated that three of the five items had a very high cross-loading, whereby they were removed. The two remaining items are presented separately. The first item, Hopefulness, “Are you hopeful about the future?” is rated on a 4-point Likert scale (1 = yes absolutely, 4 = no, not at all) and the second item, Future goals, “Do you think that you will reach your future goals?” is rated on a 3-point Likert scale (1 = yes, absolutely, 3 = no, not at all).

We initially also included measures of depression-, and anxiety symptoms together with quality of life through the patient health questionnaires *Patient Health Questionnaire (PHQ-9*), *General Anxiety Disorder (GAD−7*) and The Brunnsviken Brief Quality of Life (BBQ). However, we decided to remove these measures after running two groups as it was too time consuming and difficult for parents to answer these questions.

#### Demographic questions

Parents answered demographic questions regarding what year they were born, gender identity, the year of arriving in Sweden, if they had lived in another country before Sweden, whether family members remained in their home country, occupation, highest completed education, and if they had participated in another parenting program. They also answered two questions regarding their ability to read a document in Swedish and talk to someone in Swedish, rated on a 4-point Likert scale (1 = not at all, 4 = very well).

#### Session reports for parents

The session reports were collected after each session and contained questions regarding if parents perceived the contents of the session as comprehensible and useful, and to what extent they felt comfortable in the group. They also answered two questions regarding their perception of the leader: “How well did the leader engage the group and facilitate for everyone to have their voice heard”, and “did the parents feel welcomed and validated by the group leaders”. The parents rated these questions on a *5-*point Likert scale. Higher values indicated positive views.

#### Session reports for group-leaders

Group-leaders rated to what extent they covered the subjects and tasks in line with the manual, followed the session structure, had sufficient knowledge for running the session, validated and actively engaged the participants, and whether parents were perceived as motivated and supportive of each other. These questions were rated on a 5-point Likert scale from 1 to 5 (with higher scores indicating better manual fidelity or greater satisfaction in leading the group). Finally, the group-leaders answered a few questions in free text format, including, “What did you like about this session? Is there anything you would like to change? Would you have needed more support to lead the session? Other comments?”

#### Translation and adaptation

Measures without available translation in any of the five languages were translated. We used The World Health Organization’ protocol [37] as a guidance to achieve conceptual equivalence of the measures that have been translated to the target languages. We could not go through all the suggested steps but conducted forward translation by authorized bilingual translators with the target language as their mother tongue. An iterative approach for translating measures was used in contrast to the commonly used backtranslation to address the issue of conceptual equivalence and comprehension of the respondent [38]. The translations were therefore checked by a bilingual psychologist or a social worker to assess the conceptual equivalence of the translations. The translations were also read by group leaders and members of the target community, where possible, to assess clarity of the language.

### Group-leader interviews

All leaders who had conducted groups were asked if they wanted to participate in a semi-structured interview that would focus on their overall experience of leading the program. All leaders gave written consent to the interviews. The interviews were conducted by telephone by a research assistant, VP (see appendix 1) but were not audio recorded. The interviews were transcribed as verbatim as possible during the interview.

### Data analyses

#### Quantitative analyses

Descriptive statistics were used to present the background of the participants and their responses to session reports. Chi-square analysis and independent samples *t*-test was used to examine the differences between the completers and drop-outs at post-test. Intention to treat as well as per protocol analyses were conducted. No imputation was performed on the single items (hopefulness and future goals), and these items are presented exclusively in the per protocol analysis. Overall, 30.2% − 47.2% of the participants did not respond to post-test questionnaires. To evaluate the missing data pattern, we used Little’s MCAR test, which yielded non-significant results, suggesting that the missing data pattern was not systematic (χ^2^(89) = 96.44, *p* =.28). Thus, we used multiple imputation method to estimate missing data [39, 40]. Following recommendations for multiple imputation in small datasets, we employed 50 imputations [41] at scale score level [42]. The changes in outcome measures were tested using pairwise *t*-test or Wilcoxon signed-ranked test for non-parametric data. Pooled *t*-test results and Cohen’s *d* effect size estimates for overtime changes are presented. The effect size (*r*) calculated from Wilcoxon signed-rank test was utilized for data that did not meet the assumption of normality.

#### Qualitative analyses

Content analysis was used to analyse the interviews [43, 44]. An inductive approach was applied, whereby categories were created from raw data without a predetermined theoretical framework. The interviews were analysed in their entirety and through an active, iterative process, with recurrent discussions regarding each step in the process among two co-authors. First, the interviews were read several times and discussed. The text from two of the interviews was then divided into meaning units, which were in turn condensed, abstracted and labelled with a code. A coding manual was created, and three interviews were read and coded in parallel by two of the authors (MV and VP). These author’s different professional backgrounds, a clinical psychologist, Ph.D. student with a focus on migrant parenting programs (MV) and a medical student with a master’s degree in public health (VP), was seen as a strength to reduce bias when conducting the qualitative analyses. VP has previous experience from research within the refugee-field but has no previous experiences of parenting programs. The coded interviews were compared, the coding manual clarified, and the remaining interviews coded. Codes were compared and the meaningful relationship between them, both latent and manifest, were explored based on similarities and differences. The tentative categories were discussed and revised after a final reading of the interviews in their entirety to ensure closeness to data. Finally, a theme was formulated based on the latent content of the categories.

### Strategies regarding integration of data

A convergent mixed methods design was applied with the same sample for the quantitative and qualitative part whereby the integration could be seen as connecting [45]. First, findings from the quantitative and qualitative parts are presented separately according to the *narrative contiguous approach* and then findings are gathered together on a theme-to-theme basis according to the *narrative weaving approach* [45]. We explored if *confirmation* of data occurs between the different data collected and where there is divergence, allowing for the *expansion* of insights in terms of delivering and receiving this parent intervention [45].

### Intervention

The parenting program is group-based and consists of four sessions, each 2.5 h long including a coffee break. Each session starts with a mindfulness exercise, homework review and a presentation of the aims of the session. The program includes exercises, discussions as well as tools to practice at home between the sessions. The group-leaders are not experts, rather facilitators, and the parents learn from each other. The program considers parents as experts when it comes to their children. During all discussions “think, pair, share” is applied. That is, parents first think on their own, then share with their neighbour, and then, if they want to, share with the rest of the group. All parents received a workbook available in their preferred language, with all the content in the program briefly summarized accompanied with space to take notes and draw. An overview of the four sessions is presented in Table 1. BK (Ph.D., psychotherapist/researcher), together with MV (Ph.D. student, clinical psychologist) and PE (Ph.D. researcher, clinical psychologist) developed the manual. MV and BK led the leader training. The intervention was conducted as a part of a larger project, the PIA project (https://www.piaprojektet.se/en-gb).

**Table 1:** Overview of the contents in the four-session parenting program

Session 1: Attachment	Session 2: Communication
Being a parent in a new country Resilience and risk-/protective factors Attachment - spending time together Things that can make the situation extra challenging: adolescent development	Communication skills Attachment - having fun together Things that can make the situation extra challenging: learning a new language

Session 3: Create an open climate within the family	Session 4:The importance of school and having a context
Family meetings and problem solving Functional analysis of parental behaviour Things that can make the situation extra challenging: parental mental health regulation of emotions	To be involved in the child’s schooling To be a part of a bigger context and get friends Future dreams and goal setting

#### Session 1

In the first session the parents gain an overview of the program and start to verbalize their feelings and thoughts on being a parent in a new country. The discussion touches upon parenting tools that can be brought from their home cultural context and others that are no longer useful in this new context. Resilience as a concept is presented, and how it relates to protective- and risk factors. A further aim is to communicate the importance of attachment and present various tools to promote attachment. The message is that adolescence can function as a new important period to strengthen the bond to the youth. Adolescence can be an overwhelming time for parents as well as teenagers, why the final aim of the first session is to be familiar with adolescent development and remaining calm in certain situations.

#### Session 2

The focus of the second session is on communication. Parents practice different communication strategies such as: validation, use of “I-message” (i.e., to communicate feelings, thoughts, or needs without blaming or criticizing each other), to stay calm in conversations, and to choose the right time and place for talking with their adolescent. During the session parents also get the opportunity to express their experiences regarding the process of learning a new language. The parents discuss and share different strategies for trying to support their own and their adolescent’s language development. Finally, the parents are encouraged to think about things to do to have fun with their child to strengthen the attachment.

#### Session 3

This session focuses on how to create an open climate within the family where the adolescent wants to share details of his/her life outside the home. A way to strengthen the familial bond and support a democratic family structure is to have family meetings. A problem-solving strategy is presented during the session which encourages creative brainstorming of solutions to the perceived problem. To understand one’s own behaviour, its causes/mechanisms and the consequences it produces, a model for functional behaviour analysis is presented and practiced during the session. One theme of the session is also to discuss how to strengthen one’s ability to take care of one’s own well-being, such as stress, anxiety, and uncertainty.

#### Session 4

The last session is about being actively involved in the adolescent’s schooling. The parents discuss their experience regarding their adolescent’s schooling, what works and what they want to change, as well as parental rights and opportunities in relation to their adolescent’s education. The session content emphasized how parents can actively support their child’s engagement in school through maintained contact with teachers, participation in school meetings with an interpreter if required, and discussing school with their child in their native language. The importance of belonging to a context is emphasized and parents get to discuss what interests they, as parents, would like to develop or strengthen. The last part of the program focuses on future dreams for the parent as well as for the adolescent.

Every session ends with a summary, a reflection from the parents, homework for the week and allowing the parents to mention something they found meaningful in the session.

## Results

### Quantitative

#### Characteristics of the participants and drop-out

Over 110 different sites were contacted for recruitment, along with the recruitment conducted by leaders within their networks. This resulted in initiation of seven parenting groups (Total *N* = 54 parents): Three groups in Dari (*n* = 18), two in Somali (*n* = 16) and one group in Arabic *(n* = 4). A group in Tigrinya also started but was cancelled due to too few participants, probably because of summer holidays. We were unable to recruit Kurmanji speaking parents. One pre-test survey in Somali was not answered as the parent first attended the program at the second session, leading to a total number of 53 respondents. Thirty-eight parents completed the whole parenting program (72%). The average participation rate across all sessions was 81% (55% to 100%).

The majority of program participants were mothers (77%). The mean age of the parents was 43 years (SD = 8.57) ranging from 26 to 62 years. Almost half of the parents (49%) had been in Sweden for less than 3 years, and the majority (79%) had family members left behind. A quarter of the parents (26%) reported they had witnessed violence before leaving their home country. A large number of the parents had no schooling (60.4%, *n* = 32), whereof a majority were mothers (*N* = 27) and from Afghanistan (N *=* 16*)*. A fifth of the sample had between 2 and 6 years of education (20.8%), 13.2% had finished high school and one attended university. 30% of the parents could not speak Swedish and 41.5% could not read a document in Swedish. A high percentage of the parents were mothers that had been housewives in their homeland or without job (58.5%), whereas others had a full- or part-time job (41.5%; i.e., 22.6% men and 18.9% women). More than half of the parents had previously taken part in at least one other parenting program (56.6%).

The drop-out analysis (completers vs. non-completers) revealed no significant differences regarding gender, age, education level, or time in Sweden. The group in Tigrinya only ran for two sessions, with just one participant present at the second session, why all post-measures from that group were missing (*n* = 3). The leaders referred to unsuitable timing of the group for the parents, approaching summer holidays. Half of the Arabic-speaking parents did not participate in the last session and did therefore not respond to the post-test (*n* = 4). All Somali speaking participants except one filled in the post-test measure (*n* = 15). Regarding the three groups in Dari, 30.8% of the participants (*n* = 8) did not take part in the last session and therefore did not answer the post-test measure.

#### Change in outcomes (Intention to treat: ITT)

The outcomes are listed in Table 2. In summary, the intent-to-treat analyses showed that parents experienced a significantly increased ability to provide a “Safe haven” for their children, with a small effect size. Further, parents experienced increased “Societal belonging”, with a moderate effect size. The changes in the other measures were in the expected direction (except for Conflict) but none of them was statistically significant.

**Table 2:** Intention-to-treat (ITT) within-group analyses using paired-samples t-test or Wilcoxon signed-rank test

	Pre		Post				
Variables	Mean (SD)	Median	Mean (SD)	Median	t(df)/Z-value	p	Cohen’*s d*/*r*
Parent Network of Relationship Inventory:							
Providing a safe haven	3.93 (0.95)	4.00	4.29 (1.31)	4.50	2.0 (282)	0.047	0.33
Providing a secure base	4.49 (0.66)	4.67	4.66 (1.46)	5.00	−1.282^a^	<.05^b^	−.18^c^
Companionship	4.35 (0.58)	4.33	4.43 (1.31)	4.63	0.41 (234)	0.685	0.08
Warm relationship	4.48 (0.87)	4.80	4.61 (1.67)	4.80	0.53 (243)	0.597	0.11
Conflict	1.73 (0.80)	1.65	1.78 (1.55)	1.75	0.12 (106)	0.902	0.04
Validation	2.60 (0.51)	2.80	2.70 (1.16)	3.00	0.58 (197)	0.566	0.11
Societal belonging	3.41 (0.87)	3.50	4.33 (1.46)	4.37	4.60 (285)	< 0.001	0.77
Efficacy to support the child’s schooling	2.73 (0.58)	2.86	2.79 (0.80)	2.71	0.46 (339)	0.649	0.09

Results based on 50 imputations. ^a^ Z-value from Wilcoxon signed-rank test. ^b^*p*-value based on Fischer’s combined test. ^c^*r* was used as effect estimate for non-normally distributed data

The assumption of normality was not met for “Secure base”. Thus, a non-parametric test (Wilcoxon signed-rank test) was applied on imputed data, which suggested a statistically significant increase from pre-test to post-test (*p* <.05).

#### Change in outcomes (Per protocol)

The outcomes are listed in Table 3. The same pattern of results emerged in the per-protocol analyses (listwise deletion of the missing cases) as in the ITT for most subscales with the exception of outcome for Secure base (using Wilcoxon signed-rank test) that emerged as non-significant and thus diverging from the results of the ITT analysis. Wilcoxon signed-rank test was also applied to evaluate the outcomes measured using single items: Hopefulness and Future goals. The results suggested no significant change in these outcomes measures, opposing the outcome of secure base in the ITT-analyses.

**Table 3:** Per protocol within-group analyses using paired-samples t-test or Wilcoxon signed-rank test

	Pre-test			Post-test					
Variables	Mean (SD)		Median	Mean (SD)		Median	t (df)/Z	p	Cohen’s *d/r*
Parent Network of Relationship Inventory:									
Providing a safe haven	3.84 (0.95)		4.00	4.37 (0.83)		4.67	3.87 (35)	< 0.001	0.65
Providing a secure base	4.49 (0.68)		4.67	4.64 (0.71)		5.00	−1.44^a^	.151^b^	− 0.24 ^c^
Companionship	4.36 (0.61)		4.33	4.50 (0.67)		4.67	−.84^a^	.402^b^	− 0.14 ^c^
Warm relationship	4.48 (0.87)		4.80	4.54 (0.74)		4.80	−1.18^a^	.239^b^	− 0.22 ^c^
Conflict	1.73 (0.76)		1.63	1.86 (1.01)		1.75	−1.86^a^	.063^b^	− 0.35 ^c^
Validation	2.60 (0.50)		2.80	2.67 (0.60)		3.00	0.50 (35)	0.622	0.08
Societal belonging	3.41 (0.87)		3.50	4.27 (0.75)		4.25	5.39 (26)	< 0.001	1.04
Efficacy to support the child’s schooling	2.83 (0.62)		2.86	2.76 (0.42)		2.71	− 0.56 (36)	0.579	− 0.09
Hopefulness	1.32 (0.78)		1.00	1.38 (0.78)		1.00	−.32^a^	.751^b^	− 0.06 ^c^
Future goals	1.43 (0.67)		1.00	1.36 (0.68)		1.00	−.58^a^	.564^b^	− 0.11 ^c^

*CI* – confidence interval around Cohen’s *d*. ^a^ Z-value derived from the Wilcoxon signed-rank test. ^b^ based on Fisher’s combination of p-values. ^c^*r* was used as effect estimate for non-normally distributed data

#### Session reports for parents

An overview of the parent session reports is presented in Table 4. The majority of the parents (80%) perceived the group as safe and friendly, and that they could share their thoughts during the first session. Virtually all parents felt safe in the group from the second and the third session. The parents consistently rated “4” or “5” regarding whether the sessions were easy to understand and the content meaningful. All parents had the experience that the group-leaders engaged the group and facilitated for everyone to have their voice heard. The average rating of the leaders across all sessions was very high. The parents felt welcomed and validated. After the intervention, all except one parent said they would recommend the program to other parents.

**Table 4:** Mean, median and range for session reports filled in by parents

Evaluation of each session	First (*N* = 36)	Second (*N* = 40)	Third (*N* = 34)	Fourth (*N* = 36)
The content of today’s session was useful for me	4.81; 5.00 (3–5)	4.94; 5.00 (1–5)	4.96; 5.0 (4–5)	4.87; 5.00 (1–5)
It was possible to understand the content of today’s session	4.30; 4.63 (1–5)	4.93; 5.00 (1–5)	4.94; 5.0 (4–5)	4.82; 5.00 (1–5)
I felt safe in the group and could say whatever I wanted (Yes %)	80%	100%	100%	97.1%

Evaluation of how group-leaders managed the groups	Mean and median score for the last session (range) (*N* = 36)			
Engaged the group and facilitated for everyone to have their voice heard	5.00; 5.00 (5)			
Made you feel welcomed and validated	4.97; 5.00 (4–5)			
Maintained structure	4.97; 5.00 (4–5)			
Would you recommend the program to other parents? (Yes %)	97.2%, “No” *N* = 1			

#### Session reports for group-leaders

All group-leaders reported they had sufficient knowledge to lead each session. For every session almost all leaders reported that they had covered all the content but 1–3 group leaders indicated a certain lack of time. No clear pattern regarding the time aspect occurred, such as a certain session or program component that was too time consuming. Consistently, group-leaders reported that they managed to keep the structure of the session, validate and engage the participants. The group-leaders consistently reported very high engagement among the parents in each of the program components. No specific component could be identified with low parental engagement. Based on the group-leaders’ free text answers, they had positive experiences of leading the program. The information from the free text answers was sparse and only used to refine the program after the study (i.e., not included in the analyses). In summary, the group-leaders seemed satisfied and proud that the program seemed valuable for the parents.

### Qualitative

The result of the qualitative content analysis of the interviews of the 11 group leaders is presented in Table 5. Based on the synthesis of all categories, one theme was produced: “Meeting parents where they’re at and building trust”. Three categories “*Components of success”*, “*Learning together”* and *“Access”*. The theme is described below, whereafter we present the categories and sub-categories that were produced in the analysis.

**Table 5:** Theme, categories, and subcategories based on interviews with the group-leaders

Theme	Categories	Sub-categories
*Meeting parents where they’re at and building trust*	*Components of success*	*The process of working together*
		*Engaged parents*
		*Highlighting benefits to encourage parental participation*
		*The need for ample time*
		*Humble and competent leaders*
		*Structures and adequate resources*
	*Learning together*	*A meaningful experience*
		*How to create a safe space*
		*Personal and professional growth*
		*Engaging with the Swedish society*
		*Employing parenting strategies introduced in the program*
	*Access*	*High quality cultural adaptation of program materials*
		*Adapting to education levels*
		*Busy lives*
		*Flexibility*
		*Reaching out to parents on their own terms*

#### Theme: meeting parents where they’re at and building trust

Running a parenting program for parents to teenagers in a new cultural context requires building trust, which is facilitated by meeting parents where they are. The intervention must be implemented on the parents’ terms and be accessible, such as being run at Swedish language classes for immigrants or cultural associations. Flexibility in where and when groups are run is important as families often have busy lives. Reaching out to parents in familiar settings, such as community centres, also requires investment in in-person communication about the program.

Accessibility needs to be strengthened through high-quality materials adapted to different education and cultural backgrounds. A collaborative, mutual learning approach helps trust develop. Group-leaders emphasised the importance of creating a safe, respectful climate that encourages openness and experimentation with parenting strategies. Through building trust, parents were encouraged to engage with Swedish society and find their role as a parent within it. Meeting people where they are and building trust takes time and requires competent leaders who can invest in establishing strong working relationship. In this way, parents may become engaged and participation becomes meaningful for both parents and leaders.

#### Category 1: components of success

This category describes key components required to run a successful parental support intervention for recently, settled immigrant parents and consists of six sub-categories: *the process of working together*, *engaged parents*, *reward*, *time*, *competent leaders*, and *resources*.

#### Sub-category1.1: the process of working together

Group-leaders reported varying experiences of working together in pairs. Many emphasized that building an effective partnership takes time. Early troubleshooting was highlighted as important to establishing a well-functioning dynamic.

#### Sub-category 1.2: engaged parents

Group-leaders reported that most parents were enthusiastic, active in sessions, and were keen to learn and practice new exercises at home. Conducting sessions in the parents’ native language facilitated participation. Parents also expressed an interest in continuing the program or meeting the group again after the program ended.

#### Sub-category 1.3: highlighting benefits to encourage parents’ participation

Leaders stressed the need to motivate parents by emphasizing the potential benefits of the program for their families and opportunities to socialize with other parents. They suggested offering parents small rewards, such as a diploma or a potluck dinner, to encourage attendance.

#### Sub-category 1.4: the need for ample time

Many group leaders wished for more time to build trust and ensure everyone had a chance to speak. About half of the leaders described either keeping the discussions shorter than they wish or extending the sessions beyond 2.5 h.

#### Sub-category 1.5: humble and competent leaders

Leaders emphasized clarifying their own role so that the parents saw them as one of the group, not teachers. The leader’s role was to support and maintain the structure, include everyone, and ensure shared growth. They highlighted that all parents experience difficulties, regardless of culture or background, and that respecting everyone’s views, listening, and summarizing parents’ views builds trust. A leader needs to be humble, respectful, and interested, giving parents space to express themselves, while sharing personal experiences to model openness and equality. Several of the group-leaders noted that prior experience with group facilitation and background in education or similar fields were valuable. Flexibility, humility, enthusiasm, and patience were identified as key leader qualities.

#### Sub-category 1.6: structures and adequate resources

Adequate resources were seen as essential for program success. Leaders recommended areas for improvement such as having more group leaders to cover for one another in case of illness, running the groups directly after leader training, having experienced leaders acting as mentors to new leaders, and better refreshments for the groups.

#### Category 2: learning together

This category describes how the parental support intervention gave both parents and group-leaders an opportunity to learn from one another and about themselves. It consists of five subcategories: *a meaningful experience*, *how to create a safe space*, *personal and professional growth*, *engaging with the Swedish society* and *employing parenting strategies introduced in the program*.

#### Sub-category 2.1: a meaningful experience

Being a group-leader was described as meaningful, enjoyable, and rewarding. Leaders highlighted the importance and relevance of the program at the familial and societal level. They emphasized learning about resilience and saw the message “one’s experiences can make you stronger” as hopeful. Parents were perceived as content, grateful and committed, which was rewarding for the leaders. Meaningfulness also arose from rich discussions in parents’ mother tongues about strengthening the relationships with their youth. Some leaders wished they had attended a similar program when they first arrived in Sweden.

#### Sub-category 2.2: how to create a safe space

To foster learning, group leaders described the importance of building *trusting relationships*. They perceived that many parents, navigating a new culture, were driven by fear, making the creation of a safe space essential. Building trust *takes time*,* and while* most leaders felt that they succeeded, some parents needed longer time to open up about their experiences in Sweden and at home.

*Clear communication* and *information* about the program helped build trust, as fear of social services was seen as a barrier to participation in the program. *Physical environme*nt, such as arranging chairs in a circle, was also mentioned as helping parents feel included.

#### Sub-category 2.3: personal and professional growth

Being a leader was considered as an opportunity for both professional and personal development. Leaders described the tools in the program as universal and reported applying parenting strategies at home with their own children. They used knowledge from previous education or group experiences and expressed a wish to share experiences and mistakes with other leaders to learn more.

#### Sub-category 2.4: engaging with the Swedish society

Leaders observed that the program supported parents’ integration by encouraging engagement with the community and their children’s schools. Parents gained knowledge of where to seek help and what rights they had as parents. Language was emphasized as key for connecting with society. Some leaders said they themselves learned more about how the Swedish society functions.

#### Sub-category 2.5: employing parenting strategies introduced in the program

Leaders described how parents gradually questioned certain cultural beliefs that may no longer fit in their new context. Parents practiced new parenting skills, spending more time with their children, communicating more openly, and strengthening their relationships. They also introduced “family meetings” for weekly planning and tried to validate their child more often. The parents reported improved relationship with their children, and leaders observed parents describing that their youth wanted to spend more time with their parents.

#### Category 3: access

This category describes the importance of accessibility when implementing a parental support intervention. This category consists of five subcategories: *high-quality cultural adaptation of program materials*, *adapting to education levels*, *busy lives*, *flexibility*, and *reaching out to parents on their own terms*.

#### Sub-category 3.1: high quality cultural adaptation of program materials

Most group leaders emphasized the need to improve translations and cultural adaptation of program materials to ensure program success. Leaders spent time translating or explaining content, including adapting it to different dialects. They suggested providing leader manuals in additional languages to make group facilitation easier.

#### Sub-category 3.2: adapting to education levels

Most group leaders found the material unsuitable for parents with limited education or literacy skills, which require extra time and patience to explain and adapt the information. They recommended simplifying the manual or using short films or pictures to explain key concepts and demonstrate different scenarios.

#### Sub-category 3.3: busy lives

Balancing the program with both parents’ and group leaders’ busy lives was described as challenging. Leaders emphasized the benefits of holding groups close to parents’ homes or in easily accessible locations, such as community centres. Adapting the time and place to parents’ schedules was seen as key to attendance. Some group leaders reported high levels of engagement, while some others found motivating attendance time-consuming, particularly calling parents before sessions to remind them.

#### Sub-category 3.4: flexibility

Several leaders highlighted flexibility as essential for ensuring attendance. They described adjusting time, place and dates based on parents’ need and planning together in advance.

Flexibility also meant adapting schedules around important cultural or religious events such as Ramadan. Finally, one leader suggested increasing flexibility in the target group to include parents of children in different ages, as many families face similar challenges.

#### Sub-category 3.5: reaching out to parents on their own terms

Reaching parents was described as the most challenging and resource-intensive aspect of the program. Leaders found in-person recruitment meetings at school and community centres most effective, while information leaflets had little impact. They suggested using word-of-mouth, testimonials from previous participants, or collaborations with schools to reach more parents. Other suggested venues included language cafés, libraries, cultural centres, municipalities, and social services. Leaders also stressed the importance of reaching parents soon after arrival in Sweden, within the first few years, when the support needs are greatest.

### Integration of results (QUANT + Qual)

**Fig. 1:**
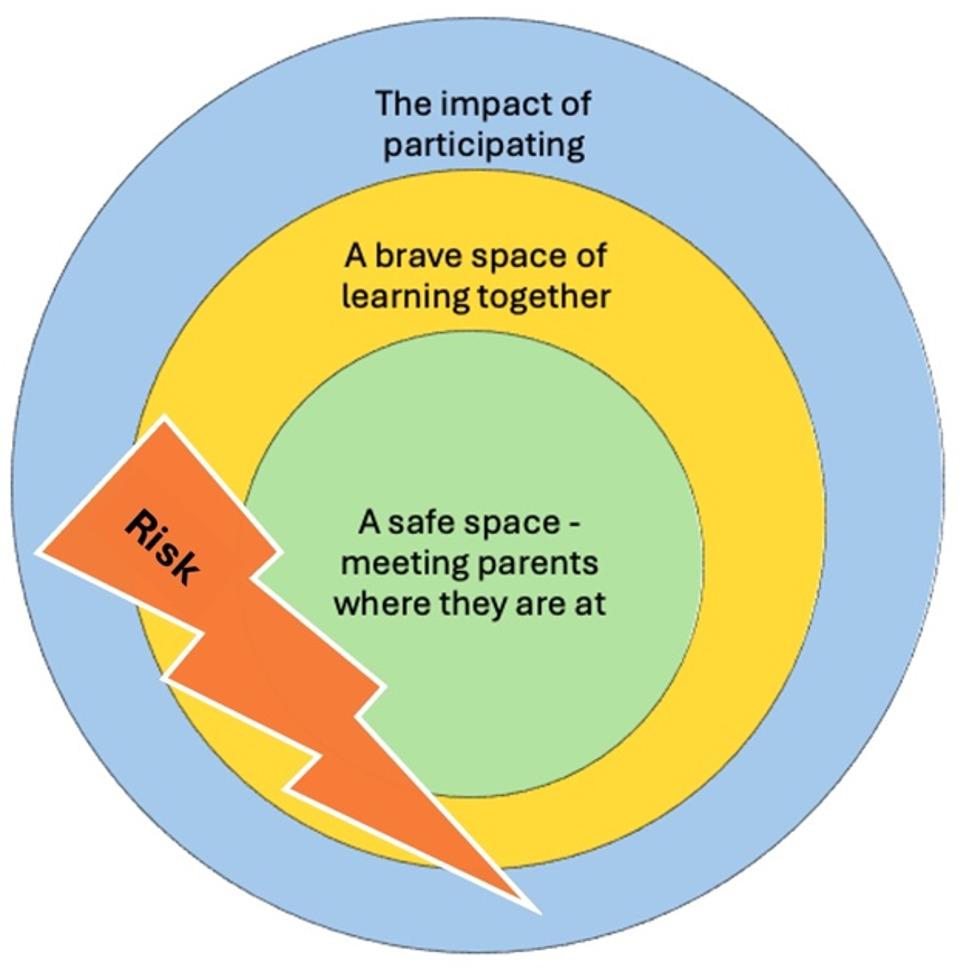
Integrated results quantitative and qualitative. Inspired from Arao and Clemens work on reframing discussions about diversity and social justice in higher education “from safe to brave spaces” [46]

The integration of the qualitative data and the quantitative data is presented in Table 6, and an illustration of the integration of the results is presented in Fig. 1.

**Table 6:** Integrated results quantitative and qualitative data

A safe space - meeting parents where they are	A brave space for learning together	The impact of participating	Risk zone
*Session reports by parents*: Experience of being welcomed, validated and safe in the group *Qual*: Access; High quality cultural adaptation of program material and adaptation to education level and reaching parents on their own terms The need of ample time, humble and capable leaders. Create trustful relationships: *clear communication* and *information and including physical environment*	*Session reports by parents*: Content as meaningful and relevant *Session reports by leaders*: Parents as active and engage during the sessions *Qual*: The process of working together as leaders together with engaged parents employing parenting strategies introduced in the program and engaging with society	*Quant*: Strengthened attachment (Safe haven) Increased feeling of societal belonging *Qual*: A meaningful experience for both leaders and parents The leaders experienced personal and professional growth	In the interviews the leaders describe parents’ busy lives as a hinder for not participating. Moreover, any element within the framework of establishing a safe space may constitute a risk factor if not adequately addressed, as not having sufficient time or inadequate cultural adaptation.

*Quant* measures, *Qual* interviews with group-leaders

#### A safe space – meeting parents where they are

Leaders emphasized that the program feasibility, acceptability, and effectiveness depend on creating a safe space and meeting parents where they are. This entails reaching parents on their own terms, having humble and capable leaders, providing culturally adapted materials, ensuring flexibility in scheduling, and allocating sufficient time and resources. Session reports from parents and interviews with leaders indicated that the program created a safe space where parents have felt welcome, validated, and secure.

#### A brave space for learning together

Parenting groups also became a *brave space*, where parents can learn together. Reports from both parents and leaders showed high engagement and positive experiences. Parents discussed parenthood across cultures and practiced various parenting skills such as validation, emotion regulation, spending quality time, and holding family meetings. The program inspired parents to engage with society, their child’s school, and their rights as parents, and to view language learning as a bridge to connection.

#### The impact of participating

Interviews and session reports indicated that participation was meaningful for both leaders and parents, fostering personal and professional growth. Both qualitative and quantitative data suggested strengthened parent-child relationships. Parents described using new skills throughout the program, which coincided with improved perception of parent-child relationship. A strengthened relationship was supported based on the improvements in parent-reported perceptions of providing a “safe haven” for their children, i.e., children seeking comfort, support, or reassurance from their parents when upset or worried. Consistently, we observed improvements in all parenting measures in expected directions except for family conflict measure.

Quantitative findings also showed increased feelings of societal belonging, supported by qualitative reports of parents growing engagement with society, schools, rights, and language learning. However, the changes in parental efficacy to engage in the child’s schooling did not reach statistical significance.

Perceived program acceptability appeared in parents session reports, where they described the program as meaningful and useful. Almost all parents said they would recommend the program to others. Consistently, leaders noted parents strong engagement in the program.

#### Risk zone

Key challenges for feasibility, acceptability, and impact included concerns with limited time and resources to create a safe space and meet parents’ needs. Both of these aspects were described as crucial for meaningful learning. Other challenges stemmed from parents’ and group-leaders’ busy lives, which could be addressed through improved flexibility. Finally, linguistic and cultural adaptations of program material were described as crucial for program success.

## Discussion and critical reflection

The results illustrated some preliminary positive effects and experiences of acceptability and feasibility from both leaders and parents.

### Preliminary outcomes

Parents’ perceptions of providing a safe haven for their youth improved significantly, albeit being a preliminary finding which should be interpreted with caution, given the non-controlled design of the study and small sample size. Providing a safe haven is associated with strong relationships within a family, an important resilience factor within a family [14]. The program included parenting strategies such as active listening, spending time together, and providing validation as ways to build stronger relationships. Similar strategies have been included in other parenting programs found effective [47]. We were keen for the program to be culturally sensitive when implementing these parental strategies. A strive was to meet parents where they are at and integrate existing parental knowledge and skills with new, as well as to build on present familial strengths, which is found as important aspects of effective interventions supporting acculturation in general [48]. Cultural sensitivity may have been facilitated as a consequence of the cultural knowledge of the group leaders and their non-expert role, in combination with the participants’ willingness to share their thoughts, questions and impressions during the group discussions.

The integration of quantitative and qualitative results suggests that the parenting program seemed to function as a brave space for the parents to openly talk about challenges, test new parenting strategies and engage in discussions. This is in line with the findings from a recent qualitative study of parenting programs for immigrant mothers with teenage children [49] where the authors stress the significance of an inclusive, non-judgmental environment that fosters cultural exchange and support. The role of the group leaders and creating a sense of security resembles some of the processes involved in attachment theory [50] where parents need to be a secure base to help the child discover its surroundings and to develop various skills. Similarly, a safe haven needed to be provided by the group-leaders to the parents, to which they can return to discuss their discoveries of new aspects of parenting in the new cultural context. The parent training groups seemed to have constituted a secure base and safe haven for parents, as reflected in the session reports from parents, and the leaders.

Parents in this study described an increased feeling of societal belonging, with a moderate effect size. Given the design of the study, it is not possible to make any firm conclusions about the actual effect. However, as the external circumstances tend to remain the same during such a brief period of intervention, it is not unlikely that the intervention might have had some impact on the perception of the parents. A potential explanation might be that the parenting groups empowered parents to actively engage in the society to a larger extent and thereby experienced increased sense of societal belonging and connectedness. Hence, empowered citizenship has been observed as an outcome of participation in a similar parenting program [49]. This includes behaviours such as actively engaging with and taking responsibility for the local community, seeking help from authorities, and adapting one’s parenting style to align with the Swedish cultural context [49]. Future studies with a control group, randomization, and collection of data on processes of change should investigate whether this effect will be replicated, and the processes that may explain this outcome.

### Acceptability

The program was well received, and appreciated based on the session reports from parents, and interviews of group leaders. Parents would recommend the program to other parents and perceived the program content as useful. The leaders described the experience of leading the program with engaged parents as meaningful, and a source of personal and professional growth. The starting point for developing the program in the current study was to examine the literature on components in universal parenting programs and to include a participatory research approach through qualitative interviews, in order to develop a culturally appropriate intervention [51]. The importance of identifying the needs of the target group together with cultural tailoring to strengthen the acceptance of the program has been highlighted in a scoping review of parenting program for immigrant families [18]. Hamari and colleagues [18] suggest that cultural tailoring should be seen as a bi-directional process including cultural tailoring from the perspective of immigrants’ cultural background, together with information about the receiving society. Further, the review also stressed the importance of cultural sensitivity and the ability to use one’s own language to ensure participation [18]. The parenting program in the current pilot study has been developed based on these principles. The program is guided by a perspective where access to multiple cultures is seen as a resource and integration as a dual responsibility of both the receiving context and resettling individual [52].

We had a large drop-out which could be an indication of low acceptability of the program. However, parents were highly positive in their ratings of the sessions and the program overall in their responses to sessions evaluations. In addition, the impression of the researchers in their interactions with participants was that they appreciated the intervention but referred to daily hassles, lack of time, or practical barriers such a no access to a babysitter to attend the sessions. These observations were confirmed by group-leader interviews where they referred to the busy lives of parents as a challenge to consistent participation in the meetings. Previous trials have addressed these barriers by for example providing child-care during the meetings and transportation to the meetings [53]. These barriers might be important to address in order to optimize the attendance and retention in the current parenting program.

### Feasibility

It seemed feasible to implement the program but it required a substantial amount of flexibility and creativity. A pragmatic approach to the intervention was crucial given specific difficulties inherent in conducting the assessments and the intervention when more than 60% of participants had no schooling. In interviews, group-leaders emphasized variables such as time, flexibility and reaching parents on their terms. To be able to meet and engage parents with such preconditions requires cultural bridges and time for building relationships (i.e., create/deserve trust). Building trust cannot be overstated when it comes to reach recently settled parents, given the distrust and fear of institutions among these parents [51], due to lack of knowledge about the traditions, customs, laws and practices in a new culture. As Schaefer et al. [54] point out, the importance of a preparatory phase to establish relationship and co-operation should be emphasized to enable research and interventions with seldom heard populations in general [54]. The components of building a safe space for parents from the integrated results could be seen as a principle to overcome potential distrust in such interventions.

During recruitment, many parents reported interest in joining the program despite having younger children than the study target group and despite residing in Sweden for a more than 6 years. Future studies should consider widening the inclusion criteria and allowing more parents to benefit from the intervention, as they are also a relevant target group.

Despite parents’ motivation and eagerness, the recruitment was the most challenging part of implementation process. This is discussed in a range of papers (e.g., [51, 55]). Although the difficulties in recruitment can be caused by many different factors, the importance of building relationships to overcome distrust, parents’ busy lives as a hinder to participate, and the stressors that they had to handle in the post-migration context were the most central factors we encountered in this study. We explored a wide range of recruitment methods and an observation throughout this project was that progress was unattainable without cultural brokers, individuals with cultural competence, and/or those possessing pre-established trust, such as teachers within Swedish for Immigrants schools. The importance of involving key individuals in the community has been stressed in previous studies as well [53]. Conventional recruitment methods in research proved ineffective in the current study. As an example, we did not receive a single interest application through our posters and flyers, and the project e-mail inbox remained empty. A multilayered approach to reach parents was necessary including top-down recruitment, to contact heads of organizations, municipalities etc. to establish the program within the organization together with bottom-up recruitment. This is in line with previous research emphasizing the need of extended timeframes, plan for higher resourcing costs and the need of operating via community partnerships [56]. In a systematic review of parent training programs for forcibly displaced families [19], all included studies used some form of community-based engagement to overcome barriers in engaging families such as collaborating with existing services, using community networks and multiple forms of active communication such as speaking in the target populations language. Furthermore, all programs were delivered in locations accessible to the parents [19] like the groups in this pilot study. The accessibility was also an important aspect emphasized in the interviews with group-leaders.

The group leaders reported that all planned session content was delivered; however, in two sessions, extended discussions created time constraints for 1–3 of the leaders. The session structure will therefore be adjusted prior to the randomized controlled trial to ensure that all content can be delivered within the designated time frame. In response to group leader feedback highlighting time constraints and for some, limited prior experience, the group leader training was also revised to include additional practice opportunities through role plays and feedback sessions to enhance delivery skills and group management.

### Strengths and limitations

A strength of the current study was the accessibility of the program in the mother tongue of recently settled parents of adolescents, to facilitate early participation in the establishment process. We also managed to reach over 60% illiterate parents through a collaboration with the group-leaders as cultural brokers. Another strength of the study was the mixed method design as they shed light on somewhat different aspects of the basically the same variables.

One potential challenge to internal validity however, at least initially, was that the manuals were not translated into the targeted languages. We tried to strengthen the implementation process of the intervention through discussions during the group-leader trainings with group-leaders on concept validity. The translated notebooks for the parents were used as a basis for discussing translations of the content and concepts in the program and their validity in for example Syrian context. Another threat to internal validity and later also external validity was the procedure of answering questionnaire when a large proportion of the parents had difficulties reading and writing. Despite effort to facilitate, for example through only having multiple choice-questions and no free text answers, as well as reading the question for the participants, this may still be a threat to the validity.

Unfortunately, some of the measures have been primarily developed and validated for Western populations. This is a common shortcoming, which may result in not capturing the intended construct [57]. We were interested in certain outcomes but few available instruments for the language groups were available. To overcome this limitation, we followed some of the steps for cultural adaptation and strived for conceptual equivalence, but we did not have the possibility to go through all the recommended steps for cultural adaptation.

Another limitation was the high dropout rate, and missingness in this study. Missing data was handled through multiple imputation, as they were not systematic. Explanation to the dropouts from the intervention was e.g. the stressful, burdensome everyday life and bad timing of the program (Ramadan and close to summer holidays).

As mentioned, we know that immigrants and especially refugees are generally underrepresented in health care seeking (e.g. [58]). We therefore need to keep in mind that the population included might reflect a group of parents more integrated in society, which may have influenced the result. We failed to reach Kurdish parents, and Tigrinya speaking parents to the same extent as Arabic-, Dari- and Somali speaking parents. We failed to identify the factors leading to no such recruitment, despite close collaboration and reoccurring discussions/problem solving with involved cultural brokers (Kurdish and Tigrinya). Finally, we didn’t succeed in including the number of parents we aimed for according to our initial plan. Despite these limitations, we found indications of acceptability and feasibility of the parent program.

## Conclusions

In the Swedish context, this is the first universal, health promoting parenting program for recently settled parents in their mother tongue that was offered in more than one language, developed from established components of parenting programs, and in accordance with parents’ experiences and perceived needs [10]. The program seems to be an acceptable intervention for recently settled immigrant parents of teenagers. To be a feasible intervention, cultural bridges and time for relation building seem crucial. The results justify further evaluation of this parent program in a randomized controlled trial.

## Supplementary Information

Supplementary Material 1

## Data Availability

The data are available from the authors upon reasonable request. Contact principal investigator: [metin.ozdemir@oru.se](mailto: metin.ozdemir@oru.se).

## Abbreviations

ACRS: Adult–Child Relationship Scale
BBQ: Brunnsviken Brief Quality of Life
FCU: Family Check-Up
GAD: General Anxiety Disorder
ITT: Intention-to-Treat
NRI-BSV: Parent Network of Relationship Inventory – Behavioral Systems Version
PHQ: Patient Health Questionnaire
Qual: Qualitative data, including interviews with group leaders
Quant: Quantitative data, including measures and session reports
STRS: Student–Teacher Relationship Scale
