# Supplementary material for: A brief universal parenting program for recently settled immigrants in Sweden: a feasibility study
Source: BMC Psychol. 2026 Jan 26;14:121. doi: 10.1186/s40359-026-04026-2 (PMC12849721; doi:10.1186/s40359-026-04026-2)
Supplement: Supplementary file 1 — Supplementary Material 1 [file 40359_2026_4026_MOESM1_ESM.docx]

Appendices

Appendix 1 – interview guide, group-leader interviews

1. Please tell me about your experiences of being a group-leader for this program.
2. What challenges do you see in leading a program like this?
3. What kind of context and environment is required to start to use (implement) this program?
4. How do you think it worked to facilitate parents’ participation in this program?
5. What is your perception of what parents gained from the sessions?
6. What benefits do you see in participating in a parenting program like this?
7. Do you have any ideas regarding how to improve the program?
8. Is there anything else you would like to express?
